# Safety of ACEi and ARB in COVID‐19 management: A retrospective analysis

**DOI:** 10.1002/clc.23836

**Published:** 2022-04-28

**Authors:** Sabina Kumar, Mastaneh Nikravesh, Umeh Chukwuemeka, Michael Randazzo, Peter Flores, Prithi Choday, Ajith Raja, Mahendra Aseri, Shah Shivang, Sumanta Chaudhuri, Pranav Barve

**Affiliations:** ^1^ Department of Internal Medicine Hemet Global Medical Center Hemet California USA; ^2^ Divison of Cardiology Loma Linda University School of Medicine Loma Linda California USA; ^3^ Division of Cardiology University of California Riverside School of Medicine Riverside California USA

**Keywords:** ACEi, ARB, COVID‐19, hypertension, mortality, safety

## Abstract

**Background & Aims:**

Severe acute respiratory syndrome coronavirus 2 (SARS‐CoV2)is a highly contagious virus that has infected 260 million individuals since December 2019. The severity of coronavirus disease 2019 (COVID‐19) depends upon the complex interplay between viral factors and the host's inflammatory response, which can trigger a cascadeeventually leading to multiorgan failure. There is contradictory evidence that angiotensin‐converting enzyme (ACEi) or angiotensin receptor blockers (ARBs) may affect mortality in patients with severe COVID‐19, theoretically due to interaction with the bradykinin pathway. Therefore, we aim to explore the association between ACEi and ARB use and mortality in severe SARS‐CoV2 infection.Severe acute respiratory yndrome with coronavirus (SARS‐CoV2) is a highly contagious virus that has infected 260 million individuals since December 2019. The severity of COVID‐19 depends upon the complex interplay between viral factors and the host's inflammatory response, which can trigger a cascadeeventually leading to multiorgan failure. There is contradictory evidence that angiotensin‐converting enzyme (ACEi) or angiotensin receptor blockers (ARBs) may affect mortality in patients with severe COVID‐19, theoretically due to interaction with the bradykinin pathway. Therefore, we aim to explore the association between ACEi and ARB use and mortality in severe SARS‐CoV2 infection.

**Materials & Methodology:**

This multicenter retrospective observational study enrolled 2935 COVID‐19 patients admitted at six hospitals in Southern California, USA, between March 2020 and August 2021. Our primary outcome was the association of pre‐hospital use of ACEi and ARB on in‐hospital mortality in COVID‐19 patients. First, relevant deidentified patient data were extracted using an SQL program from the electronic medical record. Then, a bivariate analysis of the relationship between ACEi and ARB use and different study variables using *χ* ^2^ and *t* test was done. Finally, we did a backward selection Cox multivariate regression analysis using mortality as a dependent variable.

**Results:**

Of the 2935 patients in the study, hypertension was present in 40.6%, and congestive heart failure in 13.8%. ACEi and ARB were used by 17.5% and 11.3% of patients, respectively, with 28.8% of patients on either medication. After adjusting for confounding variables in the multivariate analysis, the use of ACEi (HR: 1.226, 95% CI: 0.989–1.520) or ARB (HR: 0.923, 95% CI: 0.701–1.216) was not independently associated with increased mortality.

**Conclusion:**

Our results are consistent with the clinical guidelines and position statements per the International Society of Hypertension, that there is no indication to stop the use of ACEi/ARB in COVID‐19 patients.

## 1 INTRODUCTION

Severe acute respiratory syndrome coronavirus 2 (SARS‐CoV2) is a highly pathogenic and contagious virus which has infected 500 million individuals since December 2019. It is known to primarily affect the respiratory system, creating flu‐like symptoms which can eventually lead to severe hypoxia and acute respiratory distress syndrome (ARDS).^1^ The severity of coronavirus disease 2019 (COVID‐19) can range depending on the complex interplay between viral factors and the host's inflammatory response which can trigger a cascade eventually leading to multiorgan failure.

According to Yang et al.,^2^ the patients with COVID‐19 with comorbidity of hypertension (HTN) had a higher death rate (10.3%) and were more critically ill (18.3%) as compared with individuals without HTN. Since the onset of COVID‐19, HTN has been found to be a major risk factor in patients.^3^ The incidence of HTN and COVID‐19 were reported by the Chinese Center for Disease Control to be 12.8% worldwide, while other studies have reported as high as 30% and 31%.^4^, ^5^ Angiotensin‐converting enzyme (ACEi) and angiotensin receptor blockers (ARB) are the most widely prescribed antihypertensives in the market.^6^ There is contradictory evidence that ACEi or ARBs may either worsen or improve COVID‐19 symptoms. The proposed mechanism is discussed below.

ACE2 is a glycoprotein that is expressed in the kidney, intestine, endothelium, lungs, and heart.^7^ It is hypothesized to bind with the spiked glycoprotein in SARS‐Cov2. Once the binding occurs, SARS‐CoV2 can cause downregulation of ACE2 resulting in increased concentrations of angiotensin II causing severe lung injury.^7^ ACEi reduce angiotensin II levels which increases the proportion of ACE‐2; ARBs increase angiotensin II levels by blocking its coupling with AT2 reception leading to upregulation of ACE‐2 in the membrane.^7^ Some studies say that the availability of ACE‐2 is directly correlated with the severe inflammatory response in COVID‐19^7^ while others claim that the free form of ACE‐2 may inactivate SARS‐CoV2 and subsequently prevent the virus from entering the lungs.^7^

We aim to explore the association between pre‐hospital use of ACEi and ARBs and mortality in patients with COVID‐19. Most of the randomized trials to date have underpowered clinical endpoints and therefore cannot comment on the safety of ACEi and ARBs on adults with COVID‐19. Our study is the largest multicenter retrospective study to date with a sample size of greater than 2900 patients commenting on prior ACEi or ARB usage on mortality in COVID‐19.

## 2 METHODS

This multicenter retrospective observational study was conducted at six hospitals in Southern California, USA. The study enrolled 2935 COVID‐19 patients admitted to six hospitals between March 2020 and August 2021. All patients were confirmed to have COVID‐19 infection through a positive PCR nasopharyngeal swab. Relevant de‐identified patient data were extracted using a structured query language (SQL) program from the electronic medical record, which included: age, gender, race, comorbidities, laboratory results on admission, date of hospital admission, date of discharge, medications they received while on admission, heart rate, and disposition at discharge. Our primary outcome was in‐hospital mortality of COVID‐19 patients who were taking ACEi or ARBs before hospitalization versus those who were not.

A univariate analysis of the independent variables was performed which included: patients' age, gender, ethnicity, marital status, comorbidities, the medication patients received while in the hospital, and laboratory results using means and percentages. Furthermore, a bivariate analysis was performed of the relationship between ACEi and ARB use and different study variables using *χ* ^2^ and *t* test, with a *p* value of 0.05 considered significant. Finally, a backward selection Cox multivariate regression analysis was performed using mortality as a dependent variable. Biologically plausible or statistically significant variables from the bivariate analysis, such as age, sex, body mass index (BMI), comorbidities, use of ACEi or ARB, intensive care unit (ICU) admission, and mechanical ventilation, were used as independent variables in the multivariate model. The effect was expressed in terms of hazards ratio, and hypothesis testing was done using a two‐sided test, and an *α* value of 0.05 indicated statistical significance. Statistical analysis was completed using IBM SPSS version 27. The study was approved by the WIRB‐Copernicus Group (WCG) institutional review board (IRB).

## 3 RESULTS

### 3.1 Descriptive statistics

Tables 1 and 2 show the breakdown of continuous and categorical variables of the study population, including length of stay, age, and BMI. The age ranged between 19 and 110 years. The group's mean age ± standard deviation (SD) was 61.46 ± 18.35. There were 1276 female patients (44%) and 1656 male patients (56%). The mean BMI of the study group was 30.05, which was characterized as obese. The mean value ± SD of the inflammatory markers, c‐reactive protein (CRP), lactate dehydrogenase (LDH), Ferritin, Troponin, and creatinine phosphokinase (CPK) were 13.79 ± 9.55, 447.40 ± 464.91, 916.13 ± 1861.28, 0.48 ± 2.40, and 715.52 ± 4262.20, respectively. HTN was present in 40.6% of the study group. ACEi or ARB use was present in 17.5% and 11.3%, respectively, with a total of 28.8% of patients on either medication. 43.8% of the study population received treatment with Remdesivir.

**Table 1:** Descriptive statistics of continuous variables

	Descriptive statistics				
	*N*	Minimum	Maximum	Median	Interquartile range
Length of stay (days)	2935	1	117	7.0	9.0
Age (years)	2935	19	110	63.0	26.0
Body mass index (kg/m^2^)	2814	13.97	83.12	28.34	8.78
C‐reactive protein (mg/dl)	2375	0.04	54.38	12.95	12.77
Lactate dehydrogenase (IU/L)	1967	55	8180	363.0	261.0
Ferritin (ng/ml)	1836	5.1	47 560.8	552.1	791.57
Troponin (ng/ml)	1788	0.01	32.78	0.04	0.06
Creatinine phosphokinase (U/L)	1184	0.04	88 961.0	106.0	235.75
Platelet (10^3^/ml)	2877	21	1176	310	186.0
White blood cell count (10^3^/ml)	2873	1.8	80.7	12.5	9.1
Potassium (meq/L)	2266	2.70	10.50	4.5	1.0
Thyroid‐stimulating hormone (IU/ml)	778	0.01	94.09	1.0	1.44
Minimum heart rate	2643	20	149	57.0	14

**Table 2:** Descriptive analysis of categorical variables

	Frequency	Percent
Gender		
Female	1276	43.6
Male	1656	56.4
Race		
White	1676	57.1
Nonwhite	1258	42.9
Expired		
No	2282	77.8
Yes	653	22.2
Ventilator use		
No	2399	81.7
Yes	536	18.3
Intensive care unit admission		
No	2392	81.5
Yes	542	18.5
Remdesivir		
No	1606	56.2
Yes	1254	43.8
Tocilizumab		
No	2748	96.1
Yes	112	3.9
Dexamethasone		
No	1107	37.7
Yes	1828	62.3
Diabetes mellitus		
No	2373	80.9
Yes	562	19.1
Hypertension		
No	1743	59.4
Yes	1192	40.6
Chronic kidney disease		
No	2348	80.2
Yes	579	19.8
Acute kidney injury		
No	2678	91.2
Yes	257	8.8
Congestive heart failure		
No	2530	86.2
Yes	405	13.8
Chronic obstructive pulmonary disease		
No	2789	95
Yes	146	5
Angiotensin‐converting enzyme inhibitors (ACEi) use		
No	2422	82.5
Yes	513	17.5
Angiotensin receptor blockers (ARB) use		
No	2602	88.7
Yes	333	11.3
ACEi or ARB use		
No	2089	71.2
Yes	846	28.8
Cardiac arrest		
No	595	83.9
Yes	114	16.1

### 3.2 Bivariate analysis

In the *t* test bivariate analysis of continuous variables, age, platelet count, and potassium were significantly associated with ACEi/ARB use. The mean age for patients on ACEi/ARB was 68.3 years compared to 58.7 for those not on ACEi/ARB (*p* < .001). The mean platelet count was 319,000/ml in those on ACEi/ARB compared to 334,000/ml in those not on ACEi/ARB (*p* = .01). Additionally, the mean potassium level was 4.8 meq/L in those on ACEi/ARB compared to 4.6 meq/L in those not on ACEi/ARB (*p* < .001) (Table 3). The length of stay (LOS) was similar for both patients with or without ACEi/ARB use, and the average LOS was 11 days (Table 3).

**Table 3:** Bivariate analysis of the relationship between continuous variables and use of ACE/ARB

	ACEi or ARB	*N*	Mean	Std. deviation	*p*value
Length of hospital stay (days)	Yes	846	10.75	10.94	.69
	No	2089	10.57	11.38	
Age (years)	Yes	846	68.27	14.55	<.001
	No	2089	58.71	19.01	
Body mass index (kg/m^2^)	Yes	812	30.54	8.31	.045
	No	2002	29.85	8.38	
C‐reactive protein (mg/dl)	Yes	708	13.57	9.33	.44
	No	1667	13.89	9.64	
Lactate dehydrogenase (IU/L)	Yes	590	454.03	486.55	.68
	No	1377	444.56	455.48	
Ferritin (ng/ml)	Yes	551	856.82	1184.83	.27
	No	1285	941.57	2085.07	
Creatinine phosphokinase (U/L)	Yes	394	486.64	2037.02	.19
	No	790	829.67	5013.18	
Platelet (10^3^/ml)	Yes	824	319.38	134.67	.01
	No	2053	334.04	145.73	
White blood cell count (10^3^/ml)	Yes	824	14.60	8.73	.94
	No	2049	14.57	8.42	
Potassium (meq/L)	Yes	690	4.80	0.91	<.001
	No	1576	4.60	0.81	
Thyroid‐stimulating hormone (IU/ml)	Yes	241	2.18	3.49	.80
	No	537	2.27	6.01	
Minimum heart rate	Yes	747	56.21	13.64	.10
	No	1896	57.17	13.73	

Abbreviations: ACEi, angiotensin‐converting enzyme inhibitors; ARB, angiotensin receptor blockers.

In the *χ* ^2^ bivariate analysis of categorical variables, race (*p* = .04), diabetes (*p* < .001), HTN (*p* < .001), acute kidney injury (AKI) (*p* < .001), chronic kidney disease (CKD) (*p* = .002), congestive heart failure (CHF) (*p* < .001), chronic obstructive pulmonary disease (COPD) (*p* < .001), and coronary artery disease (CAD) (*p* < .001) were significantly associated with pre‐hospital ACEi/ARB use. Patients with diabetes (17.7% vs. 15.7%), HTN (63.5% vs. 31.4%), AKI (12.2% vs. 7.4%), CKD (23.4% vs. 18.3%), CHF (22% vs. 10.5%), COPD (7.1% vs. 4.1%), and CAD (88.9% vs. 57.7%) were more likely to be on ACEi or ARBs compared to those who were not. In addition, mortality was higher for patients on ACEi or ARB compared to those who were not (27% vs. 20.3%) (*p* < .001) (Table 4). Furthermore, in the subgroup analysis mortality was higher in patients on ACEi (28.7%) compared to those on ARB (24.3%) (Table 5).

**Table 4:** Bivariate analysis of the relationship between categorical variables and use of ARB/ACEi

	Use of ARB/ACEi		
Variable	No	Yes	*p* value
Gender			
Male	1181 (56.5%)	475 (56.1%)	.84
Female	908 (53.5%)	371 (43.9%)	
Race			
White	1164 (55.7%)	512 (60.5%)	.04
Asian	113 (5.4%)	52 (6.1%)	
Black	74 (3.5%)	34 (4.0%)	
Others	737 (35.3%)	248 (29.3%)	
Expired			
Yes	425 (20.3%)	228 (27.0%)	<.001
No	1664 (79.7%)	618 (73.0%)	
Ventilator use			
Yes	377 (18.0%)	159 (18.8%)	.63
No	1712 (82.0%)	687 (81.2%)	
Intensive care unit admission			
Yes	369 (17.7%)	173 (20.4%)	.08
No	1719 (82.3%)	673 (79.6%)	
Diabetes mellitus			
Yes	328 (15.7%)	234 (27.7%)	<.001
No	1761 (84.3%)	612 (72.3%)	
Hypertension			
Yes	655 (31.4%)	537 (63.5%)	<.001
No	1434 (68.6%)	309 (36.5%)	
Chronic kidney disease			
Yes	382 (18.3%)	197 (23.4%)	.002
No	1702 (81.7%)	646 (76.6%)	
Acute kidney injury			
Yes	154 (7.4%)	103 (12.2%)	<.001
No	1935 (92.6%)	743 (87.8%)	
Congestive heart failure			
Yes	219 (10.5%)	186 (22.0%)	<.001
No	1870 (89.5%)	660 (78.0%)	
Chronic obstructive pulmonary disease			
Yes	86 (4.1%)	60 (7.1%)	<.001
No	2003 (95.9%)	786 (92.9%)	
Coronary artery disease			
Yes	1205 (57.7%)	752 (88.9%)	<.001
No	884 (42.3%)	94 (11.1%)	
Cardiac arrest			
Yes	68 (14.7%)	46 (18.6%)	.17
No	394 (85.3%)	201 (81.4%)	

Abbreviations: ACEi, angiotensin‐converting enzyme inhibitors; ARB, angiotensin receptor blockers.

**Table 5:** Mortality in the ACEi and ARB groups

	ACEi	ARB	None	*p* value
Expired				
Yes	147 (28.7%)	81 (24.3%)	425 (20.3%)	<.001
No	366 (71.3%)	252 (75.7%)	1664 (72.9%)	

Abbreviations: ACEi, angiotensin‐converting enzyme inhibitors; ARB, angiotensin receptor blockers.

In the Kaplan−Meier bivariate survival analysis model (Figure S1), patients on ACEi had increased mortality compared with those not taking ACEi (*p* = .003). However, there was no statistically significant difference in mortality between patients who received ARBs and those who did not (*p* = .171) (Figure S2).

### 3.3 Multivariate analysis

Backward selection Cox‐regression multivariate analysis was performed to determine whether ACEi/ARB use was independently associated with mortality (Table S6). In the multivariate analysis, the use of ACEi was not independently associated with mortality (HR: 1.226, 95% CI: 0.989–1.520). Similarly, the use of ARB was also not associated with mortality (HR: 0.923, 95% CI: 0.70 –1.216).

### 3.4 Subgroup analysis

Multivariate subgroup analysis was performed to investigate other patient demographic factors or comorbidities which may be independently related to mortality in patients who received ACEi/ARB. Similar to our primary analysis, our multivariate subgroup analysis showed that ACEi/ARB use was not associated with mortality in the subset of patients with CHF and those without CHF. Similarly, ACEi/ARB use was not associated with mortality in patients with CKD versus those without CKD, and those aged 65 and above versus those below 65.

## 4 DISCUSSION

During the COVID‐19 pandemic, most of the patients who required hospitalization already had pre‐existing comorbidities such as CAD and HTN. Per Suleyman et al.'s^8^ recent case−control study, 94% of their inpatient study population diagnosed with COVID‐19 had at least one comorbidity, including HTN (63.7%), CKD (39.3%), and diabetes (38.4%). This is also supported by Martins‐Filho et al.'s^9^ study where diabetes, HTN, CKD, CAD, CHF, and COPD were noted as possible risk factors for mortality from COVID‐19.

The two studies cited above were similar to our study population in terms of respective proportion of comorbidities. According to Martin‐Filho et al.'s^9^ meta‐analysis of COVID risk factors for mortality, the male sex accounts for an increased risk of in‐hospital death due to COVID‐19. In our study, males accounted for 61.3% of in‐hospital COVID‐19 deaths with a significant *p* value of 0.005 in the bivariate analysis (Table S7).

According to Peterson et al.'s^10^ retrospective cohort study on CAD association with COVID‐19 mortality, CAD alone was not associated with mortality. However, it was noted that CAD patients affected by COVID‐19 also had higher rates of other comorbidities.

After CAD, HTN was the second most prominent comorbidity in our study. The prevalence of HTNincreases with age. The prevalence was 22.4% among adults aged 18−39 and increased to 54.5% among those aged 40−59, and 74.5% among those aged 60 years and over.^6^ In fact, high blood pressure was a primary or contributing cause of death for 516,955 people in the United States in 2019.^11^

Many of the patients with comorbidities were already on antihypertensive medications such as ACEi/ARBs before hospitalization.^9^ As it relates to COVID‐19, patients with HTN were found to be more critically ill than those without HTN.^12^ The prevalence of ACEi/ARB use in treating the patients with the above comorbidities suggests that the interactions of ACEi/ARB use in SARS‐CoV2 outcomes are worth studying.

Considered a minimal risk but vital to treatment; ACEi/ARBs required further study, but in our review of the current literature, most of the randomized trials created have been underpowered in the sample size of adults with COVID‐19. Therefore, we developed the largest multicenter retrospective study to date with a sample size of more than 2900 patients. We studied the outcomes in COVID‐19 patients who received pre‐hospital ACEi or ARBs  versus those who did not, in order to show an association with in‐hospital mortality between these two groups.

We found that ACEi/ARB use was not independently associated with mortality. Our results remained consistent in our subgroup analyses stratified by age and comorbidities including HTN, CAD, CHF, and CKD.

Our findings are overall similar to prior studies, which did not find any association between the use of ACEi/ARB and mortality. For example, we looked at the results of Huang et al.^12^, Mancia et al.^13^, and Reynolds et al.^14^. A report on 50 hospitalized patients in China found no difference in clinical characteristics between ACEi and ARBs, suggesting little effect on clinically significant severe symptoms of COVID‐19.^12^ In a large, population‐based study of 6272 COVID‐19 patients from Italy, they found that ACEi and ARB had no significant association with the risk of COVID‐19 disease.^13^

Similarly, another study in New York reported no association between ACEi and ARBs and either an increased likelihood of a positive or of severe illness.^14^ A new meta‐analysis led by the International Society of Hypertension presented at the recent American Heart Association Scientific Sessions 2021 found that randomization of COVID‐19 patients to renin‐angiotensin system (RAS) inhibitors versus controls had no effect on short‐term all‐cause mortality (7.2% vs. 7.5%, relative risk 0.95; 95% CI: 0.69−1.30). This is the largest pooled analysis to date, and included 14 randomized controlled trials and 838 patients, and the first to directly compare ACEi versus ARBs.^15^ Furthermore, the International Society of Hypertension explained the importance of continuing to take the medications as prescribed, due to the cardiovascular benefits and minimal risk. ^15^

However, the study with US Veterans by Li et al.^16^ found that the use of ACEi, but not of ARB, was associated with significantly increased odds of using mechanical ventilators; OR = 1.265, (1.010, 1.584) and OR = 1.210, (1.053, 1.39) among all COVID‐19 patients. This is interesting because our Kaplan−Meier bivariate survival analysis ‐ which did not adjust for confounding variables ‐ demonstrated increased mortality in patients on ACEi, which was not demonstratedfor patients on ARBs. Thus switching from high dose ACEi to ARB or other antihypertensives may help avoid ACEi‐triggered harmful enhancement of severe bronchoalveolar BK‐related hyperinflammation.^17^ One possible explanation is that the increased mortality seen with ACEi use was confounded by age, pre‐existing HTN, and CHF, which are associated with both ACEi/ARB use and severe COVID‐19.

Interestingly, some studies have demonstrated a decrease in mortality among patients on ARBs alone. While the clinical evidence that ARBs may limit pulmonary damage is scarce, Mortensen et al. studied 11,498 patients with pneumonitis, and showed mortality at 30 days was 13% in the total population, 30% with ACEi, and 4% with ARBs. ARB treatment during hospital stay was associated with lower mortality.^18^ Additionalclinical studies are underway to investigate this trend further.

Our study was limited by the retrospective cohort design. All patients with documented COVID‐19 infection from six Southern California hospitals were initially included in our study. The data collection was dependent on the results manually entered into the EMR, which may have caused discrepancies among the clinical variables. The limitations of using an administrative database include clerical error, limited precision of codes to describe the condition, or omission of comorbidity codes by billing or coding staff perceived irrelevant.^19^

Furthermore, we cannot completely elucidate all the factors that impact the pathway from exposure to outcome between ACE/ARB use and death. Ourconclusion may have been drawn upona fallacy which overadjusts   for factors that are not a part of the relationship between exposure and outcome. ^20^

While it is possible that there is increased mortality with ACEi use alone, that conclusion requires further research. Larger sample size and more studies are needed to achieve statistical significance to comment on the effects of ACEi alone. The lack of significance in the multivariate analysis could be due to an over‐adjustment bias of confounders.^20^ In terms of hospital mortality, it is possible that we were unable to accurately reflect risk since the length of hospitalization may be a variable dependent on ACEi or ARB as well as comorbid conditions which could lead to ACEi or ARB prescriptions. The absence of long‐term mortality is also a limitation.

The authors looked individually into pharmacy records to see if the patient was taking either ACEi or ARB before admission; however, the dose and duration were not included in the analysis. In addition, the discontinuation, addition, or change in these drugs were not taken into consideration. Additionally, ACEi/ARB use could be related to the clinical severity of the COVID‐19 infection; therefore, a future analysis could exclusively study ICU patients.

More importantly, the mechanisms of bradykinin in COVID‐19 are not fully understood, especially the pathophysiology of bradykinin. Studying the course of bradykinin and specifically its interaction with organs from exposure to ACEi/ARBs would help illustrate its true effect. Without this information, the true mortality risk cannot be demonstrated.

There are currently two approved drugs that target the kinin system: icatibant (bradykinin 2 receptor) and monoclonal antibody lanadelumab. Van deVeerdonk et al.^21^ investigated whether treatment with bradykinin 2 receptor antagonist icatibant could be used as a treatment strategy. Loss of ACE2 could lead to plasma leakage and activation of the plasma kallikrein‐kinin system, which raises bradykinin levels.^21^ The case−control study found evidence of an association between icatibant use and improved oxygenation, suggesting a benefit if given the medication in the early stages of disease when patients are hypoxic and admitted to the hospital.^21^ Van de Veerdonk as well as a clinical trial in Cleveland are also investigating the use of lanadelumab in COVID‐19 patients with pneumonia. Van de Veerdonk is currently usingmass spectrometry to measure kallikreins in the plasma of COVID‐19 patients. There are some limitations, as plasma kallikreins have a very short half‐life of around two hours.

Future studies should focus on the effects of ACEi/ARBs with concomitant use of Icatibant and Lanadelumab. It would be ideal to separate those taking ACEi  from those taking ARBs to accurately determine the effect of each drug independently. Previous use of ACEi/ARBs could potentially downregulate ACE2, therefore, minimizing the effects of Icatibant and Lanadelumab. However, without a larger sample size to cross‐reference pre‐exisiting use of ACEi/ARBs, it is difficult to truly quantify the benefits of these drugs.

### 4.1 Conclusion

Our results are consistent with the clinical guidelines and position statements; that there is no indication to stop ACEi/ARB in COVID‐19 patients. This is consistent with the International Society of Hypertension's 14 RCT meta‐analysis by Gnanethiran et al. which showed no difference in all‐cause mortality between subjects receiving ACEI/ARB and those receiving the placebo.

## CONFLICTS OF INTEREST

The authors declare no conflicts of interest.

## Supporting information

Supporting information.

## Data Availability

The data that support the findings of this study are available from the corresponding author upon reasonable request.
