# Supplementary material for: Safety of ACEi and ARB in COVID‐19 management: A retrospective analysis
Source: Clin Cardiol. 2022 Apr 28;45(7):759–66. doi: 10.1002/clc.23836 (PMC9110920; doi:10.1002/clc.23836)

Supplemental Table 6: Cox multivariate regression analysis of predictors of mortality in COVID-19 patients

|  | B | SE | Wald | Sig. | Hazard ratio | 95.0% CI for hazard ratio | |
| --- | --- | --- | --- | --- | --- | --- | --- |
|  |  |  |  |  |  | Lower | Upper |
| Age | .034 | .004 | 90.966 | 0.000 | 1.035 | 1.028 | 1.042 |
| ICU | -1.699 | .101 | 282.329 | 0.000 | 0.183 | .150 | .223 |
| BMI | .013 | .005 | 6.179 | 0.013 | 1.013 | 1.003 | 1.023 |
| REMDESIVIR | .247 | .092 | 7.123 | 0.008 | 1.280 | 1.068 | 1.534 |
| Minimum heart rate | .030 | .004 | 71.980 | 0.000 | 1.030 | 1.023 | 1.038 |
| No ACEI or ARB |  |  | 4.404 | 0.111 |  |  |  |
| ACEi | .204 | .110 | 3.461 | 0.063 | 1.226 | .989 | 1.520 |
| ARB | -.080 | .140 | .324 | 0.569 | .923 | .701 | 1.216 |
|  |  |  |  |  |  |  |  |

Supplemental Table 7: Bivariate Analysis of Gender and Mortality

| Variable | Expired | | P-value |
| --- | --- | --- | --- |
|  | No | Yes |  |
| **Gender** |  |  |  |
| Male | 1,256 (55%) | 400 (61.3%) | 0.005 |
| Female | 1,026 (45%) | 253 (38.7%) |  |

Supplemental Figure 1: Kaplan Meier for COVID-19 patients and length of stay that received ACEi and those that did not


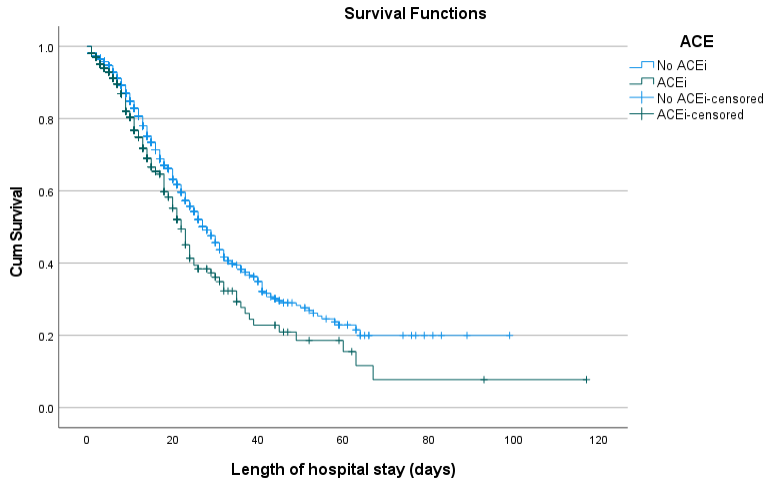


Supplemental Figure 2 Kaplan Meier for COVID-19 patients and hospital stay that received ARB’s and those that did not


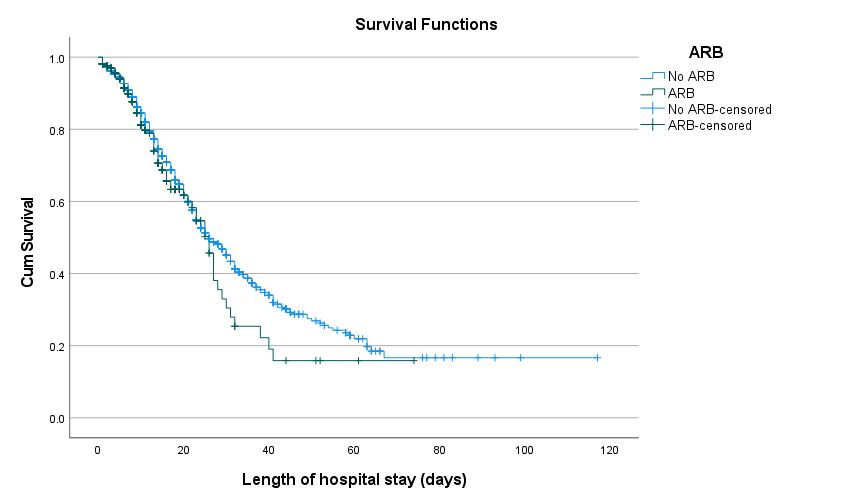

Supplement: Supplementary file 1 — Supporting information. [file CLC-45-759-s001.docx]
